# Association of Subjective Social Support With Antidepressant Treatment Response Among Older Depressed Adults in the Neurobiology of Late-Life Depression Study

**DOI:** 10.1016/j.osep.2025.08.002

**Published:** 2025-09-11

**Authors:** Ajit R. Deshpande, Rong Wu, David C. Steffens

**Affiliations:** a Department of Psychiatry, University of Connecticut School of Medicine, Farmington, CT; b Biostatistics Center, The Cato T. Laurencin Institute for Regenerative Engineering, University of Connecticut School of Medicine, Farmington, CT

**Keywords:** late-life depression, subjective social support, depression treatment outcomes, Neurobiology of Late-Life, Depression (NBOLD) Study

## Abstract

**Objectives:**

Lack of subjective social support is associated with a variety of significant public health conditions that particularly affect older adults. We sought to examine the effects of subjective social support on treatment outcomes of late-life depression (LLD).

**Methods:**

Participants in the Neurobiology of Late-Life Depression (NBOLD) Study provided self-reports regarding social support and loneliness. Study psychiatrists evaluated depressive symptoms using the Montgomery–Åsberg Depression Rating Scale (MADRS) and provided treatment with standard antidepressants. Multiple linear regression analysis examined associations between social support subscales and depression treatment outcomes, controlling for age, race, gender, education, and initial MADRS score, with the primary outcome being a change in MADRS score.

**Results:**

Among the 80 depressed participants, higher subjective social support was associated with a greater MADRS score decline over the 3-month treatment period.

**Conclusions:**

Subjective social support is significantly associated with antidepressant treatment response to LLD, highlighting a possible key factor in the management of depression in older adults.

## INTRODUCTION

Connecting social support with mental and physical health conditions has been a source of significant interest, particularly in the context of social disconnectedness arising in the wake of the COVID-19 global pandemic. Social support influencing physical and mental health factors is a well-documented concept within literature.^1^ However, when social support is parsed out more finely, the effects of its various components of have demonstrated heterogeneous or surprising findings. For instance, greater social network size was associated with greater number of reported depressive symptoms.^2^ This contrasts with literature demonstrating how limitations in both instrumental and perceived social support have been associated with longer time to remission of depression and decreased response to treatment.^3^

This heterogeneity highlights the complexity of social support as a construct, as it consists of multiple components that may have distinct and variable impacts on health outcomes, and serves as impetus for further examination into this concept in the context of treatment outcomes of late-life depression (LLD), as depression in older adults is a significant health concern that previously is adversely affected by limitations in subjective social support, a component of social support previously defined as “perceived shortage in 1’s social resources, such as companionship or social support”.^4^

Subjective social support has been associated with significant physical and mental health correlates where objective measures have not. Previous literature demonstrates how subjective perception of social support was consistently linked to better mental health, with actual received support or social integration not found to be related with health.^5^ Additionally, previous literature additionally highlights how perceived deficits in social support is associated with more severe depressive symptoms in older adults and adversely affect response to treatment of depression.^6,7^

Given renewed interest in social factors affecting mental health, particularly in older adults, it is clinically compelling to clarify the effects of social support on late-life depression treatment, specifically antidepressant treatment response, and to examine whether influences of social support, particularly subjective social support, relate to response to treatment for depression. We examined these questions using longitudinal data from the Neurobiology of Late-Life Depression (NBOLD) study. Participants in NBOLD completed measures of both objective and subjective social support. Based on prior literature, we hypothesized that a negative perception of 1’s social support would be associated with a diminished response to depression treatment with medication.

## METHODS

### Participants

Participants were enrolled in NBOLD, a National Institute of Mental Health-supported study at the University of Connecticut Health Center.^8^ Inclusion criteria for NBOLD were age 60 and older, ability to read and write in English, having a baseline Mini Mental State Examination (MMSE) score of 25 or greater, and, for the depressed cohort, meeting DSM-V-TR criteria for a major depressive episode and having a Montgomery-Asberg Depression Rating Scale (MADRS) score of 15 or higher. Exclusion criteria included current or recent alcohol use disorder, drug dependence, conditions associated with MRI abnormalities such as hydrocephalus, brain tumors, epilepsy, Parkinson’s disease, Huntington’s chorea, dementia, or demyelinating disorders. Individuals with acute endocrine disorders other than diabetes mellitus were also excluded, as were those with a physical, intellectual, or neurocognitive disability that may affect completion of self-rating instruments. Participants were also excluded if there was presence of another primary psychiatric disorder such as panic disorder, social phobia, obsessive-compulsive disorder, schizophrenia, or schizoaffective disorder. Finally, participants receiving treatment with fluoxetine were excluded due to its long washout period.

### Assessments/Measures

Depression was assessed by the MADRS as administered by a study psychiatrist at the beginning of the study, with a follow-up assessment conducted after 3 months. Social support was assessed at intake using the Duke Depression Evaluation Schedule (DDES), which included Duke Social Support Index (DSSI), a validated measure of social support with both objective and subjective metrics related to social network and connectivity.^8^ The DDES assessed social support utilizing a 36-item assessment. This allowed for the assessment of 4 subscales: social network, social interaction, subjective social support, and instrumental support.

### Standard Treatment

Upon enrollment, the study psychiatrist established a diagnosis of major depression and offered antidepressant treatment to participants, following a standard treatment protocol.^8^ Participants were initially offered treatment with sertraline 50 mg daily, or 25 mg daily for individuals aged 80 years or older. Dose increases were determined every 2 weeks as needed, up to a maximum daily dose of 200 mg. Those with a history of poor response or toleration of sertraline were offered treatment with desvenlafaxine or bupropion, or if not appropriate, would work with the study psychiatrist to identify appropriate antidepressant.

### Statistical Analysis

All the analyses were performed with SAS 9.4 using a 2-sided significance level of 0.05. Multiple linear regression was used to examine the association between social support subscales and 3-month MADRS change, controlling for age, gender, race, education and baseline MADRS, with the outcome variable being the change in MADRS Score.

## RESULTS

The sample consisted of 80 older depressed individuals with baseline MADRS scored of 15 or greater. The sample was 63.8% female, was 87.5% White, had a mean age of 71.5 years (+/− 6.5 years), and a mean education years of 15.8 (+/− 2.4 years). The population had a mean MADRS of 22.2 (+/− 4.3) at baseline and a mean MADRS score of 11.5(+/− 7.5) at 3 months, resulting in a mean change of −10.7(+/−7.9).

Table 1 shows results of multiple linear regression models of subjective social support on 3-month change in MADRS, controlling for age, gender, race, education and baseline MADRS. Both unadjusted and adjusted subjective social support were associated with 3-month MADRS change (B [95% CI]= −0.65 [−1.12, −0.18] for unadjusted version, =−0.66 [−1.17, −0.15] for adjusted version), indicating that those with higher subjective social support achieved a greater decline in MADRS.

## CONCLUSIONS

In this longitudinal study of depressed older adults, low levels of subjective social support were associated with less improvement in depression severity despite treatment while other subscales reflecting size of support networks, interpersonal interactions, and instrumental support within the DSSI were not statistically associated with depression treatment outcomes. This is consistent with prior evidence demonstrating that the subjective social support may moderate treatment outcomes in depression among older adults,^9^ as well as other studies that demonstrate that people with depression report significantly higher subjective lack of social support despite no significant difference in frequency of social contact by the participants.^10^

While subjective social support was significantly associated with treatment response to late-life depression, this association was not seen with those subscales assessing objective markers of social interactions or instrumental supports. As such, the addressing the effect of social support on late life depression treatment is unlikely to be ameliorated by simply increasing social supports for an individual but rather necessitate interventions targeting the perceived disconnect from social support.

Limitations in this study include a relatively small sample size with a study group of 80. This may result in an inability to elucidate more subtle associations, for which repeating this methodology with a larger cohort would be beneficial. Assessment of subjective social support was only conducted at intake, limiting ability to identify changes in the concept over time. Additionally, the demographics of the subjects, particularly the lack of significant racial and ethnic diversity, as well as a sample with a relatively high average level of education, limit generalizability of our findings. Further limitations include the ability to account for certain moderators and confounders, such as the heterogeneity of antidepressant regimen within the cohort. As highlighted by previous literature, our findings might be explained by adherence to treatment, as 1 prior study noted lower adherence rates to medication in older adults who report low social support. In these individuals, their beliefs regarding their control over their own illness served as a moderator of adherence.^9^

Future directions should examine targeting subjective social support as points of intervention in the overall treatment of late life depression. One potential avenue of addressing a lack of perceived social support might consider the relationship between subjective social support and loneliness. Given that the assessment of subjective social support within the DSSI includes only a single item related to loneliness, our main analyses did not focus on loneliness. However, because of the possibility that loneliness may in part explain our findings, in results not shown, we did note a significant association between loneliness and perceived social support. Thus, experience of loneliness could be seen as the emotional outcome of a deficit in an individual’s trust in their social network’s ability to meet their needs, irrespective of their ability actual level of interactions or instrumental support provided. Additionally, incorporation of an evidence-based intervention via psychotherapy may be examined to determine the effect on treatment response with intervention on subjective social support. Further studies are warranted to determine if incorporation of treatments targeting improving social support, and perhaps loneliness, would be beneficial in the overall management of LLD.

## Figures and Tables

**TABLE 1.** Regression Model for 3-Month Change in MADRS Score

Outcome: Three-Month Change (Month 3 – Baseline) in MADRS Score (N = 80)						
	Model 1 F(6, 73) = 3.65, p = 0.0032, R^2^=0.23			Model 2 F(6, 73) = 3.50, p = 0.0042, R^2^ = 0.22		
	
	Coefficient B(SE^c^)	t Value	P Value	Coefficient B(SE)	t Value	P Value
MADRS^b^, baseline	−0.76 (0.20)	−3.84	0.0003	−0.76 (0.20)	−3.81	0.0003
Subjective support, baseline	−0.65 (0.24)	−2.73	0.0080	-	-	-
Subjective Support (adjusted^a^), baseline	-	-	-	−0.66 (0.25)	−2.58	0.012
Age, years, baseline	0.06 (0.13)	0.49	0.62	0.06 (0.13)	0.45	0.66
Gender, female (reference = male)	1.26 (1.72)	0.74	0.46	1.11 (1.72)	0.64	0.52
Educational level, years	0.51 (0.34)	1.50	0.14	0.52 (0.34)	1.50	0.14
Race, Non-White (reference = White)	−1.72 (2.94)	−0.58	0.56	−1.45 (2.94)	−0.49	0.62

a Question directly inquiring about loneliness removed.

b MADRS: Montgomery–ÅSBERG depression rating scale.

c SE: standard error.

## Highlights

- What is the primary question addressed by this study?
  The question addressed in this study is how subjective social support is associated with outcomes in the treatment of late-life depression.
- What is the main finding of this study?
  Lower levels of subjective social support in older adults receiving treatment for late-life depression were associated with more severe depression at the 3-month milestone when controlling for other variables. Additionally, lower levels of subjective social support were associated with reported loneliness.
- What is the meaning of the finding?
  Understanding this association allows for identifying and intervening on subjective social support with the goal to improve treatment outcomes of late-life depression.

## References

[1] Tiikkainen P, Heikkinen RL: Associations between loneliness, depressive symptoms and perceived togetherness in older people. Aging Mental Health 2005; 9(6):526–534;doi:10.1080/13607860500193138 16214700

[2] George LK, Blazer DG, Hughes DC, : Social support and the outcome of major depression. Br J Psychiat: J Mental Sci 1989; 154:478–485;doi:10.1192/bjp.154.4.478 2590779

[3] Steffens DC, Pieper CF, Bosworth HB, : Biological and social predictors of long-term geriatric depression outcome. Int Psychogeriatr 2005; 17(1):41–56;doi:10.1017/S1041610205000979 15948303

[4] Cho JH, Olmstead R, Choi H, : Associations of objective versus subjective social isolation with sleep disturbance, depression, and fatigue in community-dwelling older adults. Aging Mental Health 2019; 23(9):1130–1138;doi:10.1080/13607863.2018.1481928 PMC6447478 30284454

[5] Uchino BN: Understanding the links between social support and physical health: a life-span perspective with emphasis on the separability of perceived and received support. Perspect Psychol Sci: J Assoc Psychol Sci 2009; 4(3):236–255;doi:10.1111/j.1745-6924.2009.01122.x 26158961

[6] Wang J, Mann F, Lloyd-Evans B, : Associations between loneliness and perceived social support and outcomes of mental health problems: a systematic review. BMC Psychiat 2018; 18 (156);doi:10.1186/s12888-018-1736-5 PMC5975705 29843662

[7] Comijs HC, Nieuwesteeg J, Kok R, : The two-year course of late-life depression; results from the Netherlands study of depression in older persons. BMC Psychiat 2015; 15(1);doi:10.1186/s12888-015-0401-5 PMC4329675 25775143

[8] Steffens DC, Manning KJ, Wu R, : Methodology and preliminary results from the neurobiology of late-life depression study. Int Psychogeriat 2015; 27(12):1987–1997;doi:10.1017/s1041610215001386 PMC4626260 26323208

[9] Bosworth HB, Hays JC, George LK, : Psychosocial and clinical predictors of unipolar depression outcome in older adults. Int J Geriatr Psychiat 2002; 17(3):238–246; doi:10.1002/gps.590 11921152

[10] Evans IEM, Llewellyn DJ, Matthews FE, : Social isolation, cognitive reserve, and cognition in older people with depression and anxiety. Aging Mental Health 2018; 23(12):1691–1700; doi:10.1080/13607863.2018.1506742 30518250

